# A revision of *Lycinella* Gorham, 1884 with the description of six new species (Coleoptera, Lycidae, Calopterini)

**DOI:** 10.3897/zookeys.792.28034

**Published:** 2018-10-23

**Authors:** Vinicius S. Ferreira, Michael A. Ivie

**Affiliations:** 1 Montana Entomology Collection, Marsh Labs, Room 50, Montana State University, Bozeman, MT 59717, USA Montana State University Bozeman United States of America

**Keywords:** Elateroidea, Leptolycini, Lycinae, Neotropical Region

## Abstract

The Neotropical genus *Lycinella* Gorham, 1884 is revised. *Lycinellaopaca* Gorham, 1884 and *Lycinellaparvula* Gorham, 1884 are redescribed and illustrated. Six new species are described for the genus: *Lycinellaadamantis***sp. n.**, *L.hansoni***sp. n.**, *L.milleri***sp. n.**, *L.cidaoi***sp. n.**, *L.marshalli***sp. n.** and *L.pugliesae***sp. n.**. *Lycinellahumeralis* Pic, 1933 is moved to *Ceratoprionhumerale* (Pic, 1933), **comb. n**. A key to the species of *Lycinella*, illustrations and a distribution map is provided.

## Introduction

While searching for cantharoid beetles in Malaise traps samples generated by the Costa Rican Malaise Trap Network project, we found a remarkable number of tiny Lycidae specimens of a unique form. These Costa Rican specimens have eight pronotal stemmata (Figure 11), an apparently unpublished character discovered by Richard S. Miller (1991) in *Leptolycus* Leng & Mutchler, 1922 (Leptolycini). However, they did not seem to belong to the Leptolycini, based on current diagnoses (Ferreira and Ivie 2016, Ferreira et al. 2018).

In discussions with Miller, he pointed us to *Lycinella* Gorham, 1884, a poorly known genus of Calopterini with three named species from Central America (Bocáková 2003, 2005) that exhibit these unreported structures. Following this lead, we found the new specimens belong to *Lycinella*, and represent several new species. In this study we take the opportunity to rediagnose and redescribe the genus and the two original species described by Gorham (*Lycinellaopaca* Gorham, 1884 and *Lycinellaparvula* Gorham, 1884), describe six new species, and provide illustrations, distribution maps and a key to all the species. One last problem was the species *Lycinellahumeralis* Pic, 1933, known in the literature only from a short description. Based on examination of the type, it is moved to *Ceratoprion* Gorham, 1884 (Leptolycini).

## Materials and methods

The specimens were examined under a Leica Wild M3C stereoscopic microscope with magnification up to 40×. Photos were taken using a JVC (DC Ky-F75U) digital camera mounted on a Leica MS5 stereoscope, a Visionary Digital Passport II imaging system, equipped with a Canon 6D DSLR (http://www.duninc.com), and a Canon T3i DSLR with a MP-E 65 mm lens and stacked using the software Zerene Stacker version 1.04. Enhancements to digital images were made in Adobe PhotoShop CC 2018. Drawings were prepared based on photographs using Adobe Illustrator CC 2018. The distribution map technique follows Ferreira (2016): the map was generated using the software Google Earth and Quantum GIS 2.18.9, using the maps available on the website http://www.naturalearthdata.com, a free public database of maps.

Morphological terminology follows Crowson (1944), Bocák and Bocáková (1990), Miller (1991), Kazantsev (2003) and Lawrence et al. (2011). Of particular note is the term “stemma” (pl. stemmata) for a unique form of structure on Leptolycini and related Calopterini, including *Lycinella*. These structures were first noted by Miller (1991), who was the first to use stemmata in this way. His usage has been subsequently followed by Ferreira and Ivie (2016) and Ferreira et al. (2018). Pronotal stemmata are tiny hemispherical white objects that occur on the pronotum, coxae and antenna of adult males (Figs 11, 12). The number and placement of these structures are diagnostic at the generic and species level. Their function and homology is unknown.

Male genitalia were dissected after the entire specimen was soaked in hot water. For disarticulation and clearing processes the specimens were left overnight in a warm solution of KOH after which they were dissected and left in cold KOH for approximately 2 hours, time enough for the musculature to detach from inner structures. Transcription of label data from specimens follows Ivie (1985): the end of each line on a label is indicated by a “;” (semicolon); the individual labels are separated by a “/” (slash).

The majority of specimens treated here were taken in the Costa Rican Malaise Trap Network project headed by Paul Hansen. Further data on this project and the localities and methods is available at Hansen (1992).

Material examined is deposited in the following collections (respective curators are indicated in parentheses):

**MAIC** Michael A Ivie collection, Bozeman, Montana, USA,

**MNCR**Museo Nacional de Costa Rica, San José, Costa Rica (the collection formerly known as INBio, Angel Solis),

**MZPW**Muzeum i Instytut Zoologii, Polskeij Akademii NaukWarszawa, Poland (David Schimrosczyk and Wioletta Tomaszewska),

**NHMUK**The Natural History Museum, London, United Kingdom (Maxwell VL Barclay and Michael Geiser),

**USNM**National Museum of Natural History, Washington D.C., USA (currently at the Montana Entomology Collection, Montana State University, Michael A Ivie).

## Results

Examination of Pic’s type showed that his *L.humeralis* belongs in the Leptolycini genus *Ceratoprion* Gorham, 1884. Therefore, we are moving it, in anticipation of a revision of the Leptolycini in progress by VF.

### Ceratoprion humerale

Taxon classificationAnimaliaColeopteraLycidae

(Pic, 1933) comb. n.

Figure 10

Lycinella humeralis Pic, 1933: 109; Kleine 1933: 34; Blackwelder 1945: 348; Mroczkowski 1959: 35. Bocák and Bocáková 1990: 667.

#### Type material examined (1).

Lectotype (hereby designated to preserve stability of nomenclature, in accordance with ICZN 1999, Art. 74.7): Costa Rica; F Nevermann; I.II.26/ Hamburgfarm; Reventazon; Ebene Limon/ Gebuseh [illegible]/ 33/ dejie [illegible]/ Lycinella; sp. det. K.G. Blair/ Lycinella; humeralis; n.n/ Typus [in a red label]/ Inst. Zool. O.A.N. Warszawa; Cotypus; Nr. 544[in a red label]/ MIZ PAN; Warszawa; 12 1945 194/ Lycinellahumeralis Pic, 1921; det V.S. Ferreira 2018 [MZPW].

#### Remarks.

Pic (1933: 109) stated that *L.humeralis* is close to *L.parvula*, but clearly differing from the latter by the longer antennae, the last antennal flagellomere in part testaceous, by the humeral portion of the elytron largely testaceous and the legs partly testaceous. Pic’s specimen lacks the diagnostic characters of *Lycinella* and possesses the characters of *Ceratoprion*: serrate antennae, reduced mandibles and strong reticulation in the elytra.

### Genus Lycinella

Taxon classificationAnimaliaColeopteraLycidae

Gorham, 1884

Lycinella Gorham, 1884: 248; Bertkau 1886: 290; Bourgeois 1891: 344; Pic 1921: 21; Kleine 1933: 34; Blackwelder 1945: 348; Bocáková 2003: 212, 230; Bocáková 2005: 445; Bocák and Bocáková 2008: 713.

#### Type species.

*Lycinellaopaca* Gorham, 1884 (subsequent designation by Bourgeois 1891: 345)

#### Differential diagnosis.

*Lycinella* can be easily identified among other Leptolycini and Calopterini by the subserrate antennae (Figs 13–20) with antennomere III longer than II but much shorter than IV, the relatively long and strongly hooked mandibles (Figure 22), the normal maxillary palps (Figure 23) and by the presence of eight discal stemmata on the pronotum (Figure 11) and stemmata on the pro- and mesocoxae (Figure 12).

#### Description.

General dorsal coloration dark brown to black, with pronotum black, yellow-brown or yellow in some species bearing dark macula in discal portion or with complete longitudinal medial region (Figs 1–9). Body densely setose, dorsal pubescence long and erect, remainder of body with fine yellow pubescence throughout (Figs 1–9).

**Figures 1–9.**
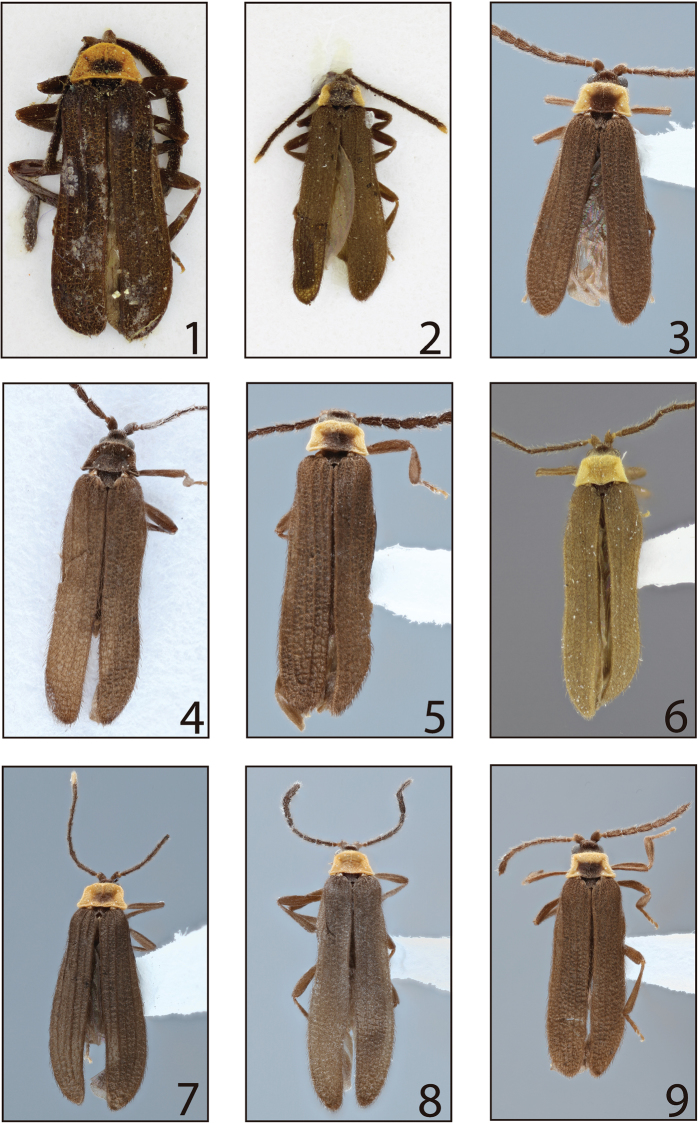
Dorsal habitus of *Lycinella*. **1***L.opaca* Gorham, 1884 (lectotype) **2***L.parvula* Gorham, 1884 (lectotype) **3***L.parvula***4***L.adamantis***5***L.hansoni***6***L.milleri***7***L.cidaoi***8***L.marshalli***9***L.pugliesae*.

Head as long as wide, widest behind eyes, posteriorly partially covered by pronotum, hypognathous. Eyes hemispherical, projecting anterolaterally when viewed dorsally; coarsely granulate. Mouthparts: Maxillary palp four-segmented, with last palpomere acuminate, densely setose (Figure 23). Labial palp 3-segmented, palpomeres I and II subequal in length, palpomere III elongate and cylindrical, acuminate, densely setose (Figure 23). Mandibles moderately enlarged to elongate, strongly hooked apically (Figure 22). Posterior margin of epistoma emarginate, labrum wider than long or longer than wide, setose (Fig. 21). Antennae inserted on gibbous prominence; subserrate to filiform; 11-segmented, with sparse short bristle-like setae on apices of antennomeres; reaching middle of elytra; scape conical to subconical, antennomere III approx. 1.5× longer than II, much shorter than IV; flagellomeres decreasing in length towards apex.

Prothorax: pronotum wider than long, trapezoidal; margins prominent; anterior angles round, posterior angles acute or moderately rounded; longitudinal carina in anterior portion of pronotum strongly to hardly visible, bifurcate posteriorly (Figs 1–9); eight pronotal stemmata located on edges of pronotum (Figs 1–9, 11). Hypomeron concave, hypomeral stemmata absent. Mesothorax: mesospiracles elongate, slightly protuberant (Figure 25). Prosternum V-shaped; posterior margin rounded to bifurcate and divergent; laterally reaching hypomeron (Figure 25). Mesoventrite trapezoidal, posteriorly reaching anterior margin of metaventrite, connected to mesanepisternum by additional segment, mesepimeron more densely pubescent than surrounding sclerites (Figure 25). Mesonotum (as represented by *L.parvula*) divided by scutellum into halves, posteriorly divergent (Figure 25); scutellum shortened, posteriorly bifurcate, of variable size (Figs 1–9; 26). Metathorax: metaventrite convex, posterolateral angles pronounced, acute; metadiscrimen complete; metanepisternum and metepimeron elongate (Figure 25–26), metendosternite (as represented by *L.parvula*) elongate, membranous, with strongly visible ventral longitudinal flange, furcal arms divergent (Figure 26). Elytra subparallel, 6–11 × longer than pronotum; reticulate, with four elytral costae more or less developed on each elytron (Figs 1–9). Membranous wings (as represented by *L.parvula*) well developed (Figure 28). Legs: slender, elongate; protrochanthin slender and exposed (Figure 27); trochanters tubular; femora and tibiae quite elongate, clavate, subequal in length (Figure 27); pro- and mesocoxae conical, moderately elongate, obliquely positioned, procoxae contiguous, some species with stemmata on each pro- and mesocoxae (Figure 12), metacoxae wider than long (Figure 25); tarsomeres 5-5-5, narrowed, tarsomere four not expanded laterally (Figure 27).

Abdomen of males with eight ventrites; male genitalia symmetrical; median lobe tapered apically to stout (Figs 29–36); parameres rounded apically (Figs 29–36); phallobase elongate to slightly shortened, with posterior margin rounded or irregular (Figs 29–36).

#### Females.

Unknown.

Length (pronotum + elytra): 3.1–4.8 mm. Width (across humeri): 0.8–1.1 mm.

#### Distribution.

*Lycinella* is known to occur in Panama, Guatemala, and Costa Rica (Figure 37).

#### Biology and immature.

Females are unknown and presumably neotenic. Although information about the ecology and biology of *Lycinella* is unknown we can infer from the fact they were virtually all taken in Malaise traps that males of *Lycinella* species are flight active species.

#### Taxonomic placement of *Lycinella*

The initial tribal placement of *Lycinella* was difficult because it is among the genera that, like *Cephalolycus* Pic, 1926 and *Aporrhipis* Pascoe, 1887, shares features of both Calopterini and Leptolycini (see Miller 1991; Bocáková 2003, 2005; Ferreira and Ivie 2016; Ferreira et al. 2018). Bocák and Bocáková (1990) placed the genus in the Leptolycini, but based on examination of *Lycinellahumeralis*, here moved to the Leptolycine genus *Ceratoprion*.

The subtribe Acroleptina (Calopterini), where *Lycinella* is currently placed, are suspected of having neotenous females (Barancikova et al. 2010), as do the known Leptolycini (Miller 1991, Kazantsev 2013, Ferreira and Ivie unpublished). Males of *Lycinella* conform to the general morphology of the groups with known or suspected neotenous females.

Ferreira and Ivie (2016) and Ferreira et al. (2018), discuss the morphological delimitation between males of Calopterini and Leptolycini, which is based on a weak tarsal character (Miller 1991, Ferreira and Ivie 2016), and placement of taxa such as *Cephalolycus*, *Aporrhipis* and Acroleptina (Ferreira and Ivie 2016, Kazantsev 2017) remains unclear. Although *Lycinella* has the narrow tarsomere IV normally present in Leptolycini, *Lycinella* lacks the reduced mouthparts found in all adult male Leptolycini. In the absence of molecular data or other evidence to the contrary, we place *Lycinella* in the Calopterini.

**Figures 10–12.**
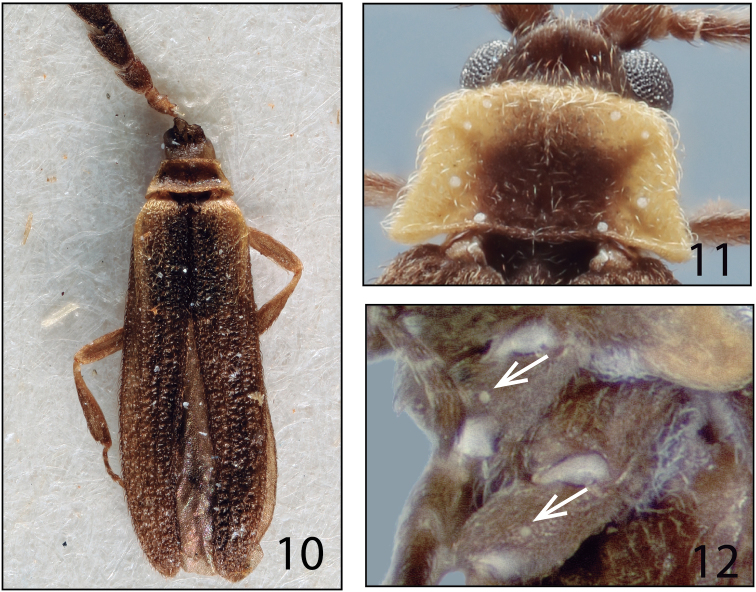
**10)** Dorsal habitus of *Lycinellahumeralis*. **11** Pronotum and pronotal stemmata of *L.parvula***12** Stemmata in pro- and mesocoxae of *L.pugliesae*.

### Key to the species of *Lycinella*

**Table:** 

1	Pronotum unicolored (Figs 4, 6, 8)	**2**
–	Pronotum bicolored (Figs 1–3, 5, 7, 9)	**4**
2	Pronotum black (Fig. 4)	***Lycinellaadamantis* sp. n.**
–	Pronotum orange or yellow (Figs 6, 8)	**3**
3	Pronotum orange (Fig. 8); phallobase with posterior margin evenly rounded, phallobase 2/3 the length of parameres (Fig. 33), labrum longer than wide	***Lycinellamarshalli* sp. n.**
–	Pronotum yellow (Fig. 6); phallobase with posterior margin irregular, phallobase 1/2 the length of parameres (Fig. 31), labrum wider than long	***Lycinellamilleri* sp. n.**
4	Elytral costae (costa I, II and III) prominent (Figs 1, 7)	**5**
–	Elytral costae weak at most, not prominent (Figs 2, 3, 5, 9)	**6**
5	Antennomeres II and XI yellow; antenna without scaliform setae (Fig. 7); dark macula in pronotal disc region faint (Fig. 7); apex of median lobe round; apex of phallobase round (Fig. 32)	***Lycinellacidaoi* sp. n.**
–	Antennomere II and XI black; antenna with scaliform setae (Fig. 1); dark macula on pronotal disc distinct (Fig. 1); apex of median lobe acuminate; apex of phallobase asymmetrical (Fig. 34)	***Lycinellaopaca* Gorham, 1884**
6	Stemmata present on pro- and mesocoxae (Fig. 12)	**7**
–	Stemmata absent on pro- and mesocoxae	***Lycinellahansoni* sp. n.**
7	Median lobe uniformly wide, not tapered apically, phallobase 1.4× shorter than parameres (Fig. 35)	***Lycinellaparvula* Gorham, 1884**
–	Median lobe tapered apically, phallobase 1.5× shorter than parameres (Fig. 36)	***Lycinellapugliesae* sp. n.**

### Lycinella adamantis

Taxon classificationAnimaliaColeopteraLycidae

Ferreira & Ivie sp. n.

http://zoobank.org/717B8819-94BA-45AB-8E0A-05496762013B

**Figures 13–24.**
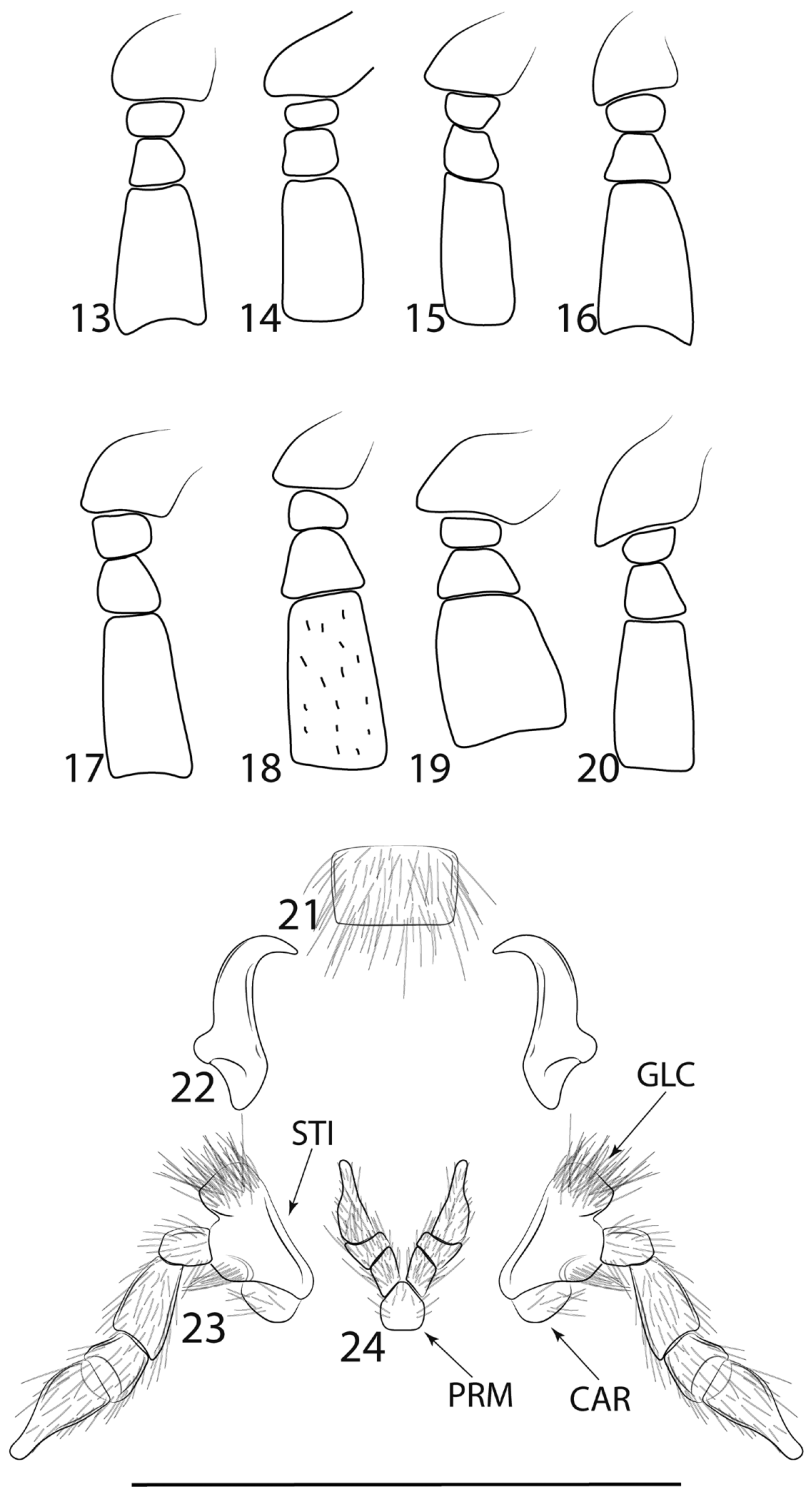
**13–20.** Antennae, antennomeres I-VI detail. **13***L.adamantis***14***L.hansoni***15***L.milleri***16***L.cidaoi***17***L.marshalli***18***L.opaca***19***L.parvula***20***L.pugliesae***21–24***Lycinellaopaca* mouthparts **21** Labrum **22** Mandibles **23** Maxillary palps **24** Labial palps. Scale bar 0.05 mm. Abbreviations: CAR: Cardo; GLC: Galea+Lacinia; PRM: Prementum; STI: Stipe.

Figs 4 , 13 , 29 , 37

#### Type material (1).

Holotype: COSTA RICA: Cartago; 4Km NE Canon, Genesis II; 9.716°N, 83.916°W; JUNE 1995, 2350m; S & P Friedman. Malaise (USNM).

#### Etymology.

The species name is in reference of the shiny pronotal stemmata that resemble small diamonds on the completely black pronotum.

#### Diagnosis.

Both the completely black body (Figure 4) and the unique male genitalia with median lobe tapered apically (Figure 29) will distinguish *L.adamantis* from all other *Lycinella* species.

#### Description.

General dorsal coloration black (Figure 4). Antennae subserrate; antennomeres IV–XI dorsoventrally flattened (Figure 13); scape subconical, antennomeres II and III short, subequal in length, approx. 1/4 length of I; antennomere IV elongate, approx. 1/3 longer than I; antennomeres V–X gradually decreasing in length; antennomere XI elongate. Mandibles elongate. Labrum wider than long. Maxillary palpomere I approx. 1/3 length of II, palpomere II cylindrical, palpomere III approx. half length of II, IV elongate, subequal in length to II, acuminate, densely setose. Labial palp 3-segmented, palpomeres I and II subequal in length, palpomere III elongate and cylindrical, acuminate, densely setose.

Pronotum trapezoidal, with posterolateral angles pronounced and acute, divergent, with weakly visible longitudinal carina in anterior portion of pronotum, bifurcate posteriorly forming an areola. Prosternum V-shaped; posterior margin rounded; laterally reaching hypomeron.

Elytra 9× longer than pronotum; costae II and IV visible, I and III weakly visible. Humeral region rounded in dorsal view. Legs slender, elongate (Figure 4). Pro- and mesocoxae with stemmata absent. Aedeagus with median lobe tapered apically, 1.4× longer than parameres; Parameres 0.6× longer than phallobase; phallobase emarginated posteriorly (Figure 29).

Length (pronotum+elytra): 4.5 mm. Width (across humeri): 1.0 mm.

#### Distribution.

Costa Rica: Cartago (Figure 37).

### Lycinella cidaoi

Taxon classificationAnimaliaColeopteraLycidae

Ferreira & Ivie sp. n.

http://zoobank.org/74CEF391-7290-4D89-B7BD-AFEAB54182BC

Figs 7 , 16 , 32 , 37

#### Type material (1).

Holotype: COSTA RICA: Alajuela; Est. Biol. Alberto Brenes; nr. San Ramon; 29 JUN 1999, 900m; MA Ivie. Malaise (USNM).

#### Etymology.

The species was named after VSF’s friend, Felipe Francisco Barbosa, a.k.a. Cidão, for his priceless advice and discussions on beetle taxonomy and systematics.

#### Diagnosis.

The weak black discal macula that does not reach any margin is unique in this species, as most other species with bicolored pronota have the strong discal macula reaching the hind margin. The exception is *L.opaca*, which has a strongly demarcated discal macula. They are further distinguished by a black antennomere XI (white in *L.cidaoi*). The unique male genitalia have a stout median lobe which is 1.3× longer than parameres and a rounded apex (Figure 32).

#### Description.

General dorsal coloration dark brown, pronotum and antennomere XI yellow, pronotum bearing weak discal black macula (Figure 7). Antennae subserrate; antennomeres IV–IX dorsoventrally flattened (Figure 16); scape subconical, antennomeres II and III short, subequal in length, approx. 1/4 length of I; antennomere IV elongate, approx. 1/3 longer than I; antennomeres V–IX gradually decreasing in length. Mandibles elongate. Labrum wider than long. Maxillary palpomere I short, approx. 1/3 length of II, which is cylindrical, palpomere III approx. half length of II, IV elongate, subequal in length of II, acuminated, densely setose. Labial palp 3-segmented, palpomere I and II subequal in length, palpomere III elongate and cylindrical, acuminated, densely setose.

Pronotum trapezoidal, not constricted medially, with posterior margin straight, anterolateral angles rounded, with posterolateral angles and pronounced and acute, divergent, with weakly visible longitudinal carina in anterior portion of pronotum, bifurcate posteriorly forming an areola, hardly visible. Prosternum V-shaped; posterior margin rounded; laterally reaching hypomeron.

Elytra approx. 11× longer than pronotum, slightly expanded in 2/3 portion; costae I, II and IV strongly visible. Humeral region rounded, non-pronounced (Figure 7). Legs slender, elongate. Pro- and mesocoxae bearing stemmata. Aedeagus with median lobe stout, apex rounded, 1.3× longer than parameres; parameres 1.3× length of phallobase; phallobase elongate with posterior margin rounded (Figure 32).

Length (pronotum+elytra): 4.5 mm. Width (across humeri): 1.1 mm.

#### Distribution.

Costa Rica: Alajuela, Biological Station Alberto Brenes (Figure 37).

### Lycinella hansoni

Taxon classificationAnimaliaColeopteraLycidae

Ferreira & Ivie sp. n.

http://zoobank.org/60C4DACA-8EC5-4FB9-878A-DA942192FFCE

Figs 5 , 14 , 30 , 37

#### Type material (1).

Holotype: COSTA RICA: Cartago; La Cangreja 1950 m; 9.8°N, 83.58°W; SEP-OCT 1992, Malaise; RA Calderon G. (USNM).

#### Etymology.

The species was described after Paul Hanson, collector of most specimens of *Lycinella* used in this study.

#### Diagnosis.

The unique genitalia of this species, characterized by the subquadrate apex of the short median lobe, which is 0.6× the length of the parameres, will distinguish this otherwise rather generalized species from all other *Lycinella* species. Among the species with a bicolored pronotum, only *L.opaca* also lacks stemmata on the pro- and mesocoxae. These two species are easily distinguished by the anteriorly rounded pronotum and strong elytral costae of *L.opaca* (Figure 1), in contrast to the angulate anterior angles and weakly costate elytra of *L.hansoni* (Figure 5).

#### Description.

General dorsal coloration dark brown, pronotum yellow, bearing longitudinal black stripe not reaching anterior margin (Figure 5). Antennae subserrate; antennomeres IV–IX dorsoventrally flattened (Figure 14); scape subconical, antennomeres II and III short, subequal in length, approx. 1/4 length of I; antennomere IV elongate, approx. 1/3 longer than I; antennomeres V–IX gradually decreasing in length. Mandibles elongate. Labrum wider than long. Maxillary palpomere I short, approx. 1/3 length of II, which is cylindrical, palpomere III approx. half length of II, IV elongate, subequal in length of II, acuminate, densely setose. Labial palp 3-segmented, palpomere I and II subequal in length, palpomere III elongate and cylindrical, acuminate, densely setose.

Pronotum trapezoidal, slightly constricted medially, with posterior margin slightly curved, anterolateral angles rounded, with posterolateral angles pronounced and acute, divergent, with weakly visible longitudinal carina in anterior portion of pronotum, bifurcate posteriorly forming an areola. Prosternum V-shaped; posterior margin rounded; laterally reaching hypomeron.

Elytra approx. 11× longer than pronotum; costae I, II and IV moderately visible (Figure 5). Humeral region rounded, non-pronounced. Legs slender, elongate. Pro- and mesocoxae not bearing stemmata. Aedeagus with median lobe uniform, apex subquadrate, 1.7× longer than parameres; parameres subequal in length of phallobase; phallobase elongate, postero-lateral angles rounded, posterior margin straight (Figure 30).

Length (pronotum+elytra): 4.2 mm. Width (across humeri): 1.0 mm.

#### Distribution.

Costa Rica: Cartago (Figure 37).

### Lycinella marshalli

Taxon classificationAnimaliaColeopteraLycidae

Ferreira & Ivie sp. n.

http://zoobank.org/81D0F2BC-0F2B-4A38-B53E-5B6111FCE58D

Figs 8 , 17 , 33 , 37

#### Type material (1).

Holotype: CR: Puntarenas, San; Gerardo de Dota, Savegre; Lodge, Canto de las Aves; trail; 19–21 FEB 2008, SA Marshall; debut00319381 (MNCR).

#### Etymology.

The species was named after Steve Marshall, who collected the specimen of this species for this study.

#### Diagnosis.

The elongate labrum, which is longer than wide, is unique among all *Lycinella* species. The pronotum solid yellow-brown is shared only with *L.milleri*, which has a short labrum. The only known male genitalia are broken (Figure 33) and so cannot be fully diagnosed.

#### Description.

General dorsal coloration dark brown, pronotum orange (Figure 8). Antennae subserrate; antennomeres IV–XI dorsoventrally flattened (Figure 17); scape subconical, antennomeres II and III short, subequal in length, approx. 1/4 length of I; antennomere IV elongate, approx. 1/3 longer than I; antennomeres V–X gradually decreasing in length; antennomere XI elongate. Mandibles elongate. Labrum wider than long. Maxillary palpomere I short, approx. 1/3 length of II, which is cylindrical, palpomere III approx. half length of II, IV elongate, subequal in length of II, acuminate, densely setose. Labial palp 3-segmented, palpomere I and II subequal in length, palpomere III elongate and cylindrical, acuminate, densely setose.

Pronotum trapezoidal, posterior margin straight, anterolateral angles rounded, with posterolateral angles pronounced and acute, divergent, with weakly visible longitudinal carina in anterior portion of pronotum, bifurcate posteriorly forming weakly visible areola. Prosternum V-shaped; posterior margin rounded; laterally reaching hypomeron.

Elytra approx. 10× longer than pronotum; costae weakly visible. Humeral region rounded (Figure 8). Legs slender, elongate. Pro- and mesocoxae without stemmata. Aedeagus with parameres 2× longer than phallobase; phallobase rounded posteriorly (Figure 33).

Length (pronotum+elytra): 4.8 mm. Width (across humeri): 1.0 mm.

#### Distribution.

Costa Rica: San Gerardo de Dota (Figure 37).

### Lycinella milleri

Taxon classificationAnimaliaColeopteraLycidae

Ferreira & Ivie sp. n.

http://zoobank.org/42FA890B-A37A-4392-800C-63B5D56EB8EB

Figs 6 , 15 , 31 , 37

#### Type material (1).

Holotype: COSTA RICA: San Jose; 19 km S., 3 Km W. Empalme; 9.650°N, 83.866°W; DEC1992, 2600m; P Hanson Malaise (USNM).

#### Etymology.

Noun, neuter. This species is named in honor of the great North American Lycidae systematist, Richard Stuart Miller.

#### Diagnosis.

The unicolorous yellow-brown pronotum is shared with only *L.marshalli*, but the stronger elytral costae of *L.milleri* (Figure 6) and its wider–than-long labrum will distinguish it easily from *L.marshalli* (Figure 8). The male genitalia is also unique, with the emarginate posterior margin of the phallobase (Figure 31) shared only with *L.publiesae* (Figure 36), which has a bicolored pronotum and rounded apex of the median lobe.

#### Description.

General dorsal coloration dark brown, pronotum orange (Figure 6). Antennae subserrate; antennomeres IV–XI dorsoventrally flattened (Figure 15); scape subconical, antennomeres II and III short, subequal in length, approx. 1/4 length of I; antennomere IV elongate, approx. 1/3 longer than I; antennomeres V-X gradually decreasing in length; antennomere XI elongate. Mandibles elongate. Labrum elongate. Maxillary palpomere I short, approx. 1/3 length of II, which is cylindrical, palpomere III approx. half length of II, IV elongate, subequal in length of II, acuminate, densely setose. Labial palp 3-segmented, palpomere I and II subequal in length, palpomere III elongate and cylindrical, acuminate, densely setose.

Pronotum trapezoidal, anterolateral angles rounded, with posterolateral angles and pronounced and acute, divergent, with weakly visible longitudinal carina in anterior portion of pronotum, bifurcate posteriorly forming weakly visible areola. Prosternum V-shaped; posterior margin rounded; laterally reaching hypomeron.

Elytra approx. 10× longer than pronotum; costae strongly visible. Humeral region rounded (Figure 6). Legs slender, elongate. Pro- and mesocoxae without stemmata. Aedeagus with median lobe tapered apically, 1.5× longer than parameres; parameres 2.5× longer than phallobase; phallobase emarginated posteriorly (Figure 31).

Length (pronotum+elytra): 3.4 mm. Width (across humeri): 0.8 mm.

#### Distribution.

Costa Rica: San José (Figure 37).

### Lycinella opaca

Taxon classificationAnimaliaColeopteraLycidae

Gorham, 1884

Figs 1 , 18 , 34 , 37

Lycinella opaca Gorham, 1884: 249 table XI, fig. 15; Bertkau 1886: 290; Bourgeois 1891: 345; Kleine 1933: 34 [in part, Panama record to L.parvula]; Blackwelder 1945: 348; Bocák and Bocáková 1990: 639; Bocáková 2003: 230 figs 19, 38, 49, 70, 71, 123–125.

#### Type material examined (2).

Lectotype and paralectotype (hereby designated to preserve stability of nomenclature, in accordance with ICZN (1999) Art. 74.7). 1♂ Lectotype: *Lycinella*; *opaca*; Gorham/ B.C.A. Col. III. (2).; *Lycinella; opaca*, Gorham/Type/ Syntype/ Type; sp. figured/ San Juan; Vera Paz.; Champion/ LECTOTYPE; Lycinellaopaca Gorham, 1884; det V.S. Ferreira 2018 (NHMUK). 1♂ Paralectotype: San Juan; Vera Paz.; Champion/ Syntype/ B.C.A. Col. III. (2).; Lycinella; opaca, Gorham/ Compared with type/ PARALECTOTYPE; *Lycinellaopaca* Gorham, 1884; det V.S. Ferreira 2018 (NHMUK).

#### Diagnosis.

The stout antennae and rounded anterior margin of the pronotum are unique to this species (Figure 1). The strong pronotal macula that does not reach the base of the pronotum is also diagnostic. The scaliform setae on antennomeres IV-XI are likewise unique in the genus (Figure 18). The male genitalia (Figure 34) can be used to confirm the identification.

#### Redescription.

General dorsal coloration dark brown, pronotum and antennomere XI yellow, pronotum bearing weak strong black macula (Figure 1). Antennae subserrate, bearing sparse scaliform setae on antennomerrs IV–XI; antennomeres IV–IX dorsoventrally flattened (Figure 19); scape subconical, antennomeres II and III short, subequal in length, approx. 1/4 length of I; antennomere IV elongate, approx. 1/3 longer than I; antennomeres V-–IX gradually decreasing in length. Mandibles elongate.

Pronotum trapezoidal, not constricted medially, with posterior margin slightly arcuate, anterolateral angles rounded, with posterolateral angles and pronounced and round, divergent, with weakly visible longitudinal carina in anterior portion of pronotum, bifurcate posteriorly forming an areola, hardly visible.

Elytra approx. 10× longer than pronotum, slightly expanded in 2/3 portion; costae I, II, and III strongly visible. Humeral region rounded, non-pronounced (Figure 1). Aedeagus with median lobe elongate, apex acuminate, twice longer than parameres; parameres half length of phallobase; phallobase elongate with posterior margin rounded (Figure 34).

Length (pronotum+elytra): 3.6 mm. Width (across humeri): 0.9 mm.

#### Distribution.

Guatemala (Figure 37).

### Lycinella parvula

Taxon classificationAnimaliaColeopteraLycidae

Gorham, 1884

Figs 2 , 3 , 11 , 19 , 25–28 , 35 , 37

Lycinella parvula Gorham, 1884: 249 table XI, fig. 16; Bertkau 1886: 290; Blackwelder 1945: 348; Bocáková 2003: 230.
Lycinella opaca not Gorham; Kleine 1933: 34 [Panama record], see Remarks below.

#### Type material examined (3).

Lectotype and paralectotypes (designated to preserve stability of nomenclature, in accordance with ICZN (1999) Art. 74.7, hereby designated). 1♂ (Lectotype): B.C.A. Col. III. (2).; Lycinella; parvula, Gorham/ Lycinella; parvula; Gorham/ Type/ Syntype/ Type; sp. figured/ Bugaba. 800–1500 ft.; Champion/ LECTOTYPE; Lycinella; parvula Gorham, 1884; det V.S. Ferreira 2018 (NHMUK). 2♂ (Paralectotypes): V. de Chiriqui; 25–4000 ft; Champion/ Syntype/ B.C.A. Col. III. (2).; Lycinella; parvula, Gorham /PARALECTOTYPE; Lycinella marginata Gorham, 1884; det V.S. Ferreira 2018(NHMUK). **Material examined in addition to type specimens (133)**: 24: COSTA RICA: Puntarenas; 24Km W. Piedras Blancas; 8°46'N, 83°24'W, 200m; DEC1991, M. Salablanca N; Malaise trap, 1°forest (MAIC). 3: COSTA RICA: Puntarenas; 24Km W. Piedras Blancas; 8.766°N, 83.400°W; NOV1991, 200m, G. Dullce; P. Hanson. Malaise (MAIC). 2: COSTA RICA: Puntarenas; 24Km W. Piedras Blancas; 8.766°N, 83.400°W; 21 NOV1991, 200m, G. Dullce; P. Hanson. Malaise (MAIC). 6: COSTA RICA: Puntarenas; 24Km W. Piedras Blancas; 8.766°N, 83.400°W; MAR-APR 1993, 200m, G. Dullce; P. Hanson. Malaise (MAIC). 1: COSTA RICA: Puntarenas; 24Km W. Piedras Blancas; 8.766°N, 83.400°W; AUG-SEP 1993, 200m, G. Dullce; P. Hanson. Malaise (MAIC). 15: COSTA RICA: Puntarenas; 24Km W. Piedras Blancas; 8°46'N, 83°24'W, 200m; JUN1991, M. Salablanca N; Malaise trap, 1°forest (MAIC). 1: COSTA RICA: Puntarenas; 5Km W. Piedras Blancas; 8°46'N, 83°17'W, 100m; JUL1991,; Malaise trap (MAIC). 19: COSTA RICA: Puntarenas; 27Km S. Puerto Jimenez; Rio Piro; Nov 1990, 75m; P. Hanson. Malaise (MAIC). 3: COSTA RICA: Puntarenas 24Km S. Puerto Jimenez; Finca La Jilba; SEP1990, 75m [1 specimen 100m]); P. Hanson. Malaise (MAIC). 1: COSTA RICA: Puntarenas; 27Km S. Puerto Jimenez; Rio Piro; Nov 1993, 75m; P. Hanson. Malaise (MAIC). 1: COSTA RICA: Puntarenas; 27Km S. Puerto Jimenez; Finca La Jilba; JUL-SEP 1993, 100m; P. Hanson. Malaise (MAIC). 7: COSTA RICA: Pr. Pedernales; Penn. de Osa; Rancho Quemedo; 2Km N. on Camino Drake; 275m, Nov 1990; Riyito colr. (MAIC). 3: COSTA RICA: Puntarenas; 24Km S. Puerto Jimenez; Finca La Jilba; JUL-SEP 1993 100m; P. Hanson. Malaise (MAIC). 3: COSTA RICA: Puntarenas; 3Km SW Rincon; 8.683°N, 83.438°W; June 1991, 10m; P. Hanson. Malaise (MAIC). 8: COSTA RICA: Puntarenas; 3Km SW. Rincon, Golfo Dulce; 8.683°N, 83.438°W; OCT-DEC1990, 10m; P.Hanson, Malaise (MAIC). 2: COSTA RICA: Puntarenas; Cerro Rincon, 200m, S. Hito; 8.516°N, 83.466°W; OCT1990, 745m, P.Hanson; & Godoy, Malaise (MAIC). 5: COSTA RICA: Puntarenas; 8KM S.Rio Rincon punte; 8.633°N, 83.466°W, 10m; APRIL 1992, Malaise trap; P. Hanson. (MAIC). 2: COSTA RICA: Pr. Puntarenas; 5Km NW Puerto Jimenez; 8°33'N, 82°21'W, 10m; APRIL 1992, Malaise trap.; P.E. Hanson colr. (MAIC). 3: COSTA RICA: Pr. Puntarenas; 3Km SW Rincon, 10m; 8°41'N, 83°29'W; AUG 1991. Malaise trap.; P.E. Hanson colr. (MAIC). 1: COSTA RICA: Puntarenas; Cerro Rincon, 745; 8.516°N 83.466°W; SEP 1990, P.Hanson; Malaise, virgin forest (MAIC). 1: COSTA RICA: Puntarenas; 5Km NW Puerto Jimenez; 8°683°N, 83.483°W; SEP 1991, 10m; P. Hanson, Malaise (MAIC). 2: COSTA RICA: Puntarenas; 23Km N. Puerto Jimenez; La Palma; JULY 1993, 10m; P.Hanson. Malaise (MAIC). 7: COSTA RICA: Puntarenas: Rancho Quemado; Rio Riyito; NOV 1990, 200m; P.Hanson, Malaise (MAIC). 1: COSTA RICA: San Jose; Zurqui de Moravia; 10°03'03"N, 84°00'22"W; MAY 1996 1600m; C. Flores. Malaise (MAIC). 1: COSTA RICA: Pr. Puntarenas; San Vito, Est. Biologica; Las Alturas, 1500m; 8°57'N, 82°50W; JUNE 1992, Malaise trap; P.E. Hanson colr. (MAIC). 1: COSTA RICA: Puntarenas; San Vito, Est. Bio. Las; Alturas, 1500m; 8.950N°, 82.833°W; OCT1991; P.Hanson, Malaise (MAIC). 1: COSTA RICA: Alajuela; Est. Biol. San Ramon; OCT-DEC 1995, 900m; P. Hansen. Malaise (MNCR). 1: COSTA RICA: Alajuela; Est. Biol. San Ramon; OCT-DEC 1995, 900m; P. Hansen. Malaise (MNCR). 1: COSTA RICA: Alajuela; Est. Biol. Alberto Brenes; nr. San Ramon; 29 JUN 1999, 900m; M. A. Ivie, Malaise (MNCR). 2: COSTA RICA: Alajuela; Est, Biol. Alberto Brenes; nr. San Ramon; JULY-AUGUST 1995, 900m; P. Hansen, Malaise (MAIC). 2: COSTA RICA: Alajuela; Est, Biol. Alberto Brenes; nr. San Ramon; AUGUST-SEPT 1995, 900m; P. Hansen, Malaise (MAIC). 1: COSTA RICA: Prov. Cartago; La Cangreja, 1950m; 9°48'N, 83°58'W; NOV 1991, Malaise trap; R.A. Calderón G. colr (MNCR). 1: COSTA RICA: Puntarenas; 24Km W. Piedras Blanas; 8°46'N, 84°24'W 200m; DEC1991, M. Salablanca N; Malaise trap, 1° Forest (MAIC). 1: Costa Rica: San Jose; P. N. Braulio Carrillo; 9.5Km E. tunnel, 1000m; 10.116°N, 83.966°W; JAN-FEB1990, P.Hanson; Malaise, Virgin Forest (MAIC).

#### Diagnosis.

The bicolored pronotum with the discal macula reaching the posterior margin places this species with *L.hansoni* and *L.pugliesae*. It can be distinguished from *L.hansoni* by the presence of stemmata on the pro- and mesocoxae (also present in *L.pugliesae* and *L.cidaoi*). It is very similar to *L.pugliesae* but is more widespread and common than that high elevation species. The male genitalia must be consulted to be sure of the identification. In *L.parvula*, the median lobe is subparallel with a truncate apex and the parameres are broadly rounded (Figure 35). In *L.pugliesae* the median lobe is constricted just past the apex of the parameres and rounded at the apex (Figure 36) and the parameres are narrowly rounded.

#### Redescription.

**Figures 25–28.**
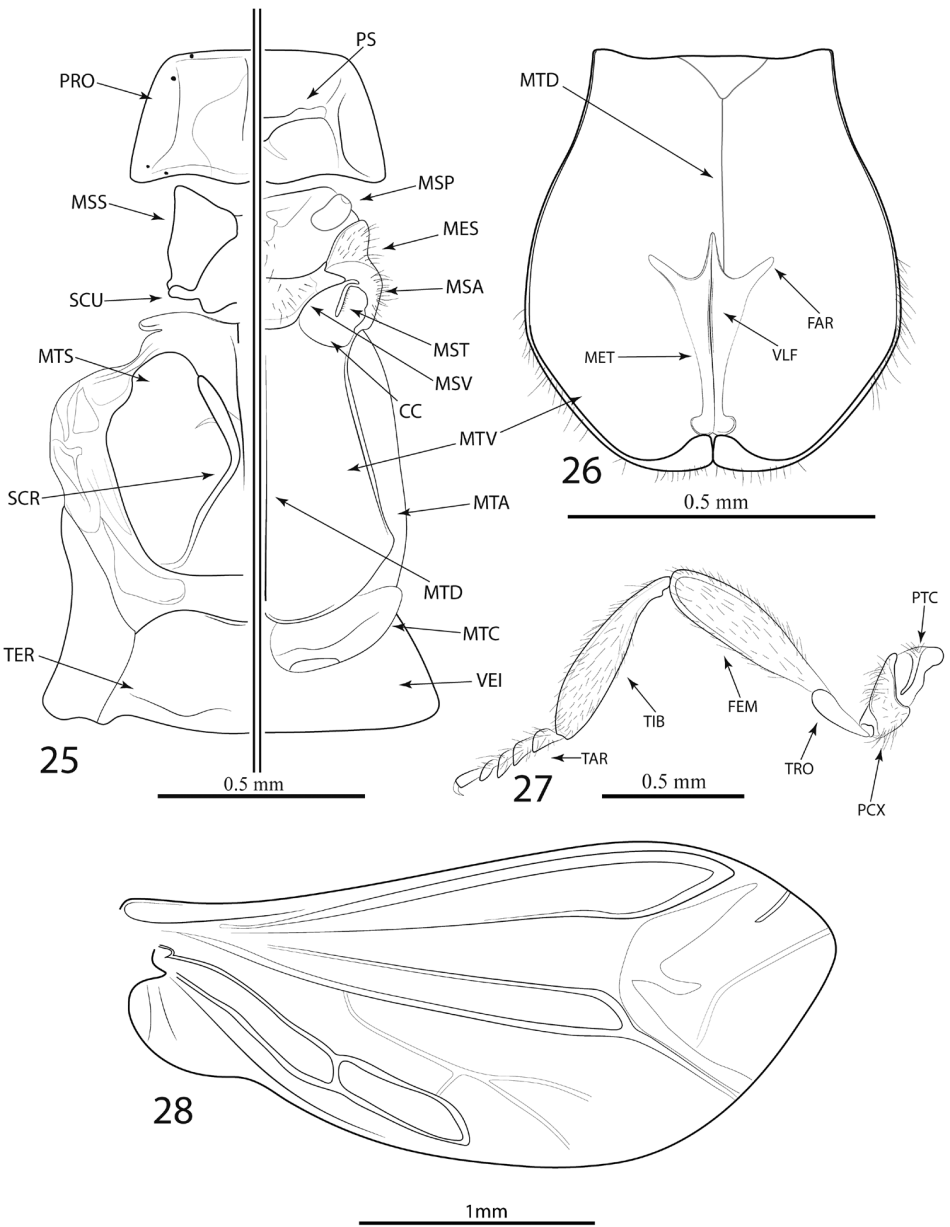
*Lycinellaparvula* morphology. **25** Thorax in ventral and dorsal view **26** Dorsal view of metaventrite and metendosternite **27** Proleg **28** Metathoracic wing. Abbreviations: CC: Coxal cavity; FAR: Furcal Arms; FEM: Femur; MES: Mesepimeron; MET: Metendosternite; MSA: Mesanepisternum; MSP: Mesoespiracle; MSS: Mesoscutum; MST: Mesotrochantin; MSV: Mesoventrite; MTA: Metanepisternum; MTD: Metadiscrimen; MTN: Metanotum; MTS: Metascutum; MTV: Metaventrite; PCX: Procoxae; PRO: Pronotum; PS: Prosternum; PTC: Protrochantin; SCR: Scutoprescutal ridge; SCU: Scutellum; TAR: Tarsi; TER: Tergite I; TIB: Tibia; TRO: Trochanter; VEI: Ventrite I; VLF: Ventral longitudinal flange.

General dorsal coloration dark brown, pronotum and antennomeres XI yellow, pronotum bearing longitudinal black stripe (Figure 9). Antennae subserrate; antennomeres IV–XI dorsoventrally flattened (Figure 19); scape subconical, antennomeres II and III short, subequal in length, approx. 1/4 length of I; antennomere IV elongate, approx. 1/3 longer than I; antennomeres V–X gradually decreasing in length; antennomere XI elongate. Mandibles elongate. Labrum wider than long. Maxillary palpomere I short, approx. 1/3 length of II, which is cylindrical, palpomere III approx. half length of II, IV elongate, subequal in length of II, acuminated, densely setose. Labial palp 3-segmented, palpomere I and II subequal in length, palpomere III elongate and cylindrical, acuminated, densely setose. Pronotum trapezoidal, anterolateral angles rounded, with posterolateral angles and pronounced and acute, divergent, with weakly visible longitudinal carina in anterior portion of pronotum, bifurcate posteriorly forming weakly visible areola (Figure 11). Prosternum V-shaped; posterior margin rounded; laterally reaching hypomeron (Figure 25).

***Elytra*** 7.5–10× longer than pronotum (Figs 2, 3); costae I, II, and III more visible. Humeral region rounded (Figs 2, 3). Legs slender, elongate (Fig. 27). Pro- and mesocoxae bearing stemmata. Aedeagus with median lobe uniform, slender, 1.4× longer than parameres; parameres 1.5× longer than phallobase; phallobase rounded posteriorly (Figure 36).

Length (pronotum + elytra): 3.2–3.5 mm. Width (across humeri): 0.8–1.0 mm.

#### Distribution.

Costa Rica and Panama (Figure 37).

#### Type locality.

Panama, Bugaba, Volcan de Chiriqui.

#### Remarks.

*Lycinellaparvula* was put in synonymy with *L.opaca* by Kleine (1933). *L.parvula* was reinstated as a valid species by Bocáková 2003.

### Lycinella pugliesae

Taxon classificationAnimaliaColeopteraLycidae

Ferreira & Ivie sp. n.

http://zoobank.org/A29ECA07-1D42-4BF4-AEA9-C5A4C7B4F492

Figs 9 , 12 , 20 , 36 , 37

#### Type material (2).

Holotype: COSTA RICA: Prov. San José; Zurquí de Moravia, 1600 m; 10°03'N, 84°01'W; APRIL 1996. cloud forest; JA Lizano, Malaise trap (USNM). Paratype: COSTA RICA: San José; Zurquí de Moravia; 10°03'03"N, 84°00'22"W; MAY 1996, 1600 m; C Flores, Malaise (MAIC).

#### Etymology.

The species was described after VSF’s former Zoology professor, Dr Adriana Pugliese Netto Lamas, which greatly influenced, inspired, and helped him in his early career as zoologist.

#### Diagnosis.

*Lycinellapugliesae* is very similar to *L.parvula*, see the diagnosis for that species for further information.

#### Description.

General dorsal coloration dark brown, pronotum yellow, bearing longitudinal black stripe not reaching anterior margin (Figure 9). Antennae subserrate; antennomeres IV–IX dorsoventrally flattened (Figure 20); scape subconical, antennomeres II and III short, subequal in length, approx. 1/4 length of I; antennomere IV elongate, approx. 1/3 longer than I; antennomeres V–IX gradually decreasing in length. Mandibles elongate. Labrum wider than long. Maxillary palpomere I short, approx. 1/3 length of II, which is cylindrical, palpomere III approx. half length of II, IV elongate, subequal in length of II, acuminated, densely setose. Labial palp 3-segmented, palpomere I and II subequal in length, palpomere III elongate and cylindrical, acuminate, densely setose.

Pronotum trapezoidal, slightly constricted medially, with posterior margin slightly curved, anterolateral angles rounded, with posterolateral angles and pronounced and acute, divergent, with weakly visible longitudinal carina in anterior portion of pronotum, bifurcate posteriorly forming an areola. Prosternum V-shaped; posterior margin rounded; laterally reaching hypomeron.

Elytra 10× longer than pronotum; costae on each elytron, costae I, II, and IV moderately visible. Humeral region rounded, non-pronounced (Figure 9). Legs slender, elongate. Pro- and mesocoxae bearing stemmata (Figure 14). Aedeagus with median lobe tapered apically, slender, apex rounded, 1.6× longer than parameres; parameres 1.5× longer than phallobase; phallobase elongate, posterior angles rounded (Figure 36).

Length (pronotum+elytra): 3.1 mm. Width (across humeri): 0.8 mm.

#### Distribution.

Costa Rica: Prov. San José, Zurquí de Moravia (Figure 37).

**Figures 29–36.**
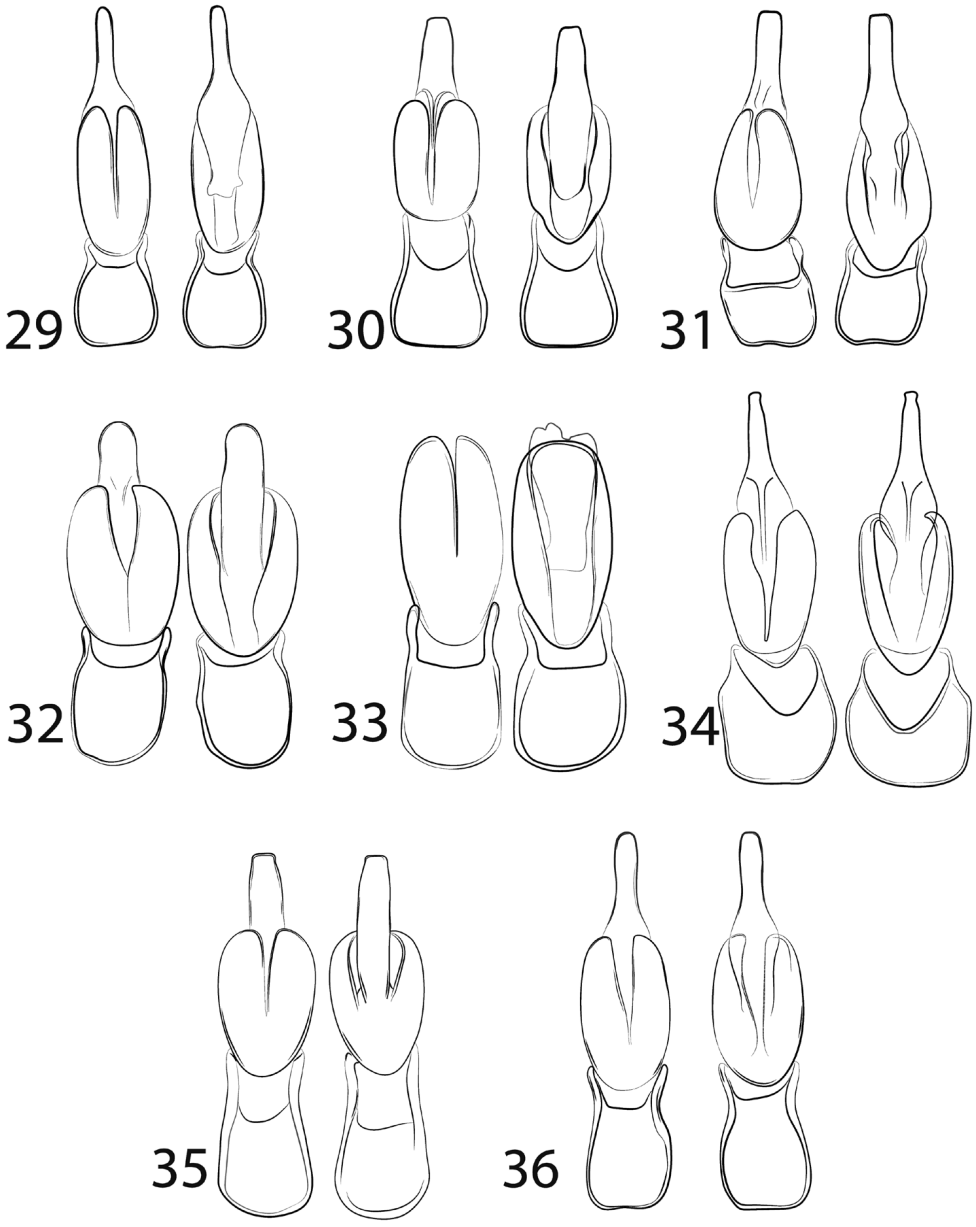
Male genitalia of *Lycinella* spp. in dorsal and ventral view. **29***L.adamantis***30***L.hansoni***31***L.milleri***32***L.cidaoi***33***L.marshalli***34***L.opaca***35***L.parvula***36***L.pugliesae*.

**Figure 37.**
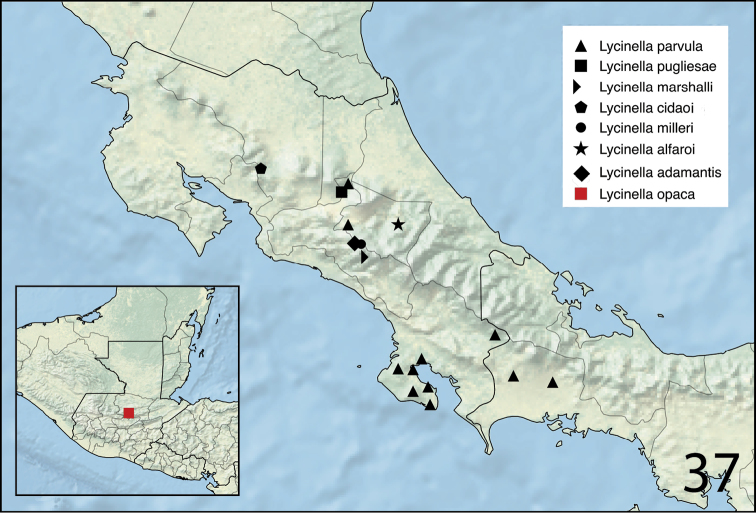
Distribution map of *Lycinella* species in Panama, Costa Rica, and Guatemala (inset map).

## Supplementary Material

XML Treatment for Ceratoprion humerale

XML Treatment for Lycinella

XML Treatment for Lycinella adamantis

XML Treatment for Lycinella cidaoi

XML Treatment for Lycinella hansoni

XML Treatment for Lycinella marshalli

XML Treatment for Lycinella milleri

XML Treatment for Lycinella opaca

XML Treatment for Lycinella parvula

XML Treatment for Lycinella pugliesae
